# Numerical Analysis of Occupant Head Injuries in Impacts with Dump Truck Panel

**DOI:** 10.1155/2018/8373479

**Published:** 2018-06-03

**Authors:** Shence Wang, Deshun Liu, Zhihua Cai

**Affiliations:** School of Electromechanical Engineering, Hunan University of Science and Technology, Xiangtan 411201, China

## Abstract

The human head will inevitably impact on the panel causing injury due to the inertia during dump truck collisions or emergency braking. Therefore, this paper aims to analyze the effects of panel design parameters on occupant head injuries via simulations using finite element (FE) models of a human head and a dump truck cockpit. Special focus was applied to understand how panel type (soft and hard), elastic modulus of the filling and frame, and the fixing distance for the soft panel could affect head injuries in head-to-panel impacts under different impact conditions (impact speed and location). Simulation results show that a soft panel is beneficial for head protection in impacts with the truck instrument panel, and a soft panel using a lower filling elastic modulus, lower frame elastic modulus, and longer fixing distance is helpful for head injury prevention. The findings also indicate that the head peak acceleration and maximum skull stress are more sensitive to the fixing distance and elastic modulus of frame than elastic modulus of the filling of the panel. Moreover, these trends are not affected by changing the impact speed and impact location. The findings of this study suggest that a safer panel design for head injury prevention should firstly have a long fixing distance and then followed by using softer filling and frame materials.

## 1. Introduction

The dump truck is widely used for transportation of large-scale metal mines, coal mines, water resources, and so on with high efficiency and loading capability. However, the safety of dump truck is particularly an important issue due to the complex workplace. Impacts between the head and instrument panel in a condition of truck collision or emergency braking generally lead to greater threat to the lives of the occupant of a dump truck. Therefore, the safety of the panel has become important for vehicle design.

At present, the study of the panel is mainly based on the United Nations Economic Commission for Europe Regulation number 21 (ECE R21 [1]). In this regulation, the head is simplified to a solid model with a rigid material body and a vinyl nonlinear material skin, and then they are bonded together as the impactor. In the tests, the diameter of the head form is 165 mm, the mass for the impactor is 6.8 kg, and the impact speed is 24.1 km/h. The ECE R21 requires that the measured acceleration response of the head form under the prescribed test condition should not exceed 80 g continuously for more than 3 ms. Physical impact test using head form is an important method for study of panel safety design. Liu et al. obtained spherical head model acceleration changes based on impact tests of head form and gravity impactor with a panel [2]. However, physical impact test is not the optimal approach for study of panel design since it is difficult to control the factors around the period of the tests and the parameters about the impact test are inconvenient to change. Moreover, the circle of the physical impact test is long and the cost is high. Therefore, numerical simulations using the head form and car models were widely used for panel safety design. Haniffah et al. ascertained the appropriate material of the panel according to the rules of the head form stress changing predicted from simulations [3]. Zhao and Zhao [4] and Bao and Qi-ming [5] optimized the panel by changing panel attribute parameters to reduce head acceleration based on simulations. However, previous studies on panel safety design were mainly based on head form as mentioned above, which cannot predict the biomechanical response of human head in the collisions.

The purpose of this study is therefore to analyze the effects of different panel design parameters on occupant head injury using a more accurate human head model via finite element (FE) simulations. In particular, the design parameters of the panel type, filling elastic modulus, frame elastic modulus, and length of panel were considered. This work will provide important data for the design of truck panel with the purpose of reducing occupant head injuries in the complex workplace [16–18].

## 2. Materials and Methods

### 2.1. Head FE Model

**Figure 1:**
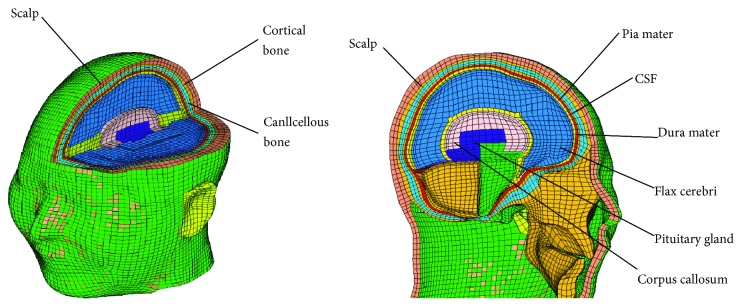
The FE model of the head.

The head FE model was extracted from a whole body model which was developed and used to study the chest injury during impacts in different directions [12]. The head model was then improved and validated by Mao et al. in a later study [13], see Figure 1. The validation study mainly focused on evaluating the biomechanical response of the head model under loading conditions of drop and blunt impact at different speeds comparing with cadaver test data from Yoganandan et al. [14, 15], and the validation results show good agreement with the experiments [13]. Thus, the head FE model is reliable for analysis in the current study.

The brain is a soft biological organ with high water content (close to 80%). It shows incompressibility, nonlinearity, anisotropy, and viscoelasticity. Thus, the ^∗^MAT_VISCOELASTIC (6# material in LS-DNYA) was selected. Plenty of brain tissue experiments show that the deformation of the brain tissue depends on its shear modulus, so its formula is as follows:

(1) $$\begin{array}{c} G\left(t\right)=G_{0}+\left(G_{0}-G_{\infty }\right)e^{-\beta t}, \end{array}$$

where *G*_0_ is short-term shear modulus, *G*_∞_ is long-term shear modulus, and *β* is decay coefficient. The material parameters used for the head model components are given in Tables 1 and 2.

### 2.2. Dump Truck Cockpit FE Model

**Figure 2:**
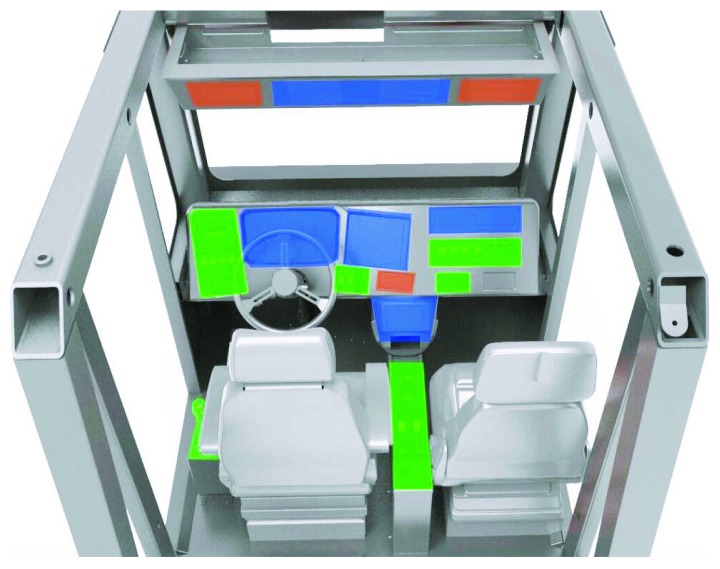
The FE model of the dump truck.

The FE model of a dump truck cockpit was developed using a finite element preprocessing software HyperMesh. It consists of the truck framework, seat, panel, and other structures in the cockpit, see Figure 2. The dump truck cockpit FE model includes 1513114 nodes and 1505251 elements.

Two panel types (soft panel and hard panel) are usually used in dump trucks. Soft panels are mainly made up of polyvinyl chloride (PVC) and polycarbonate (ABS), while hard panels are usually made of plastics. For the soft panel, ABS and PVC materials are usually used in the skin and frame, and the polyurethane (PUR) material is chosen as the filing layer to reduce the stiffness of the panel.  Table 3 shows the materials for the truck framework and the panel, where the DC01 low carbon steel was selected to model the frame of dump truck and the material models for the soft and hard panel were defined, respectively. The data in Table 3 were extracted from a previous study of head-to-panel impacts [4].

### 2.3. Setup of Impact Simulations

#### 2.3.1. Impact Location and Impact Speed

**Figure 3:**
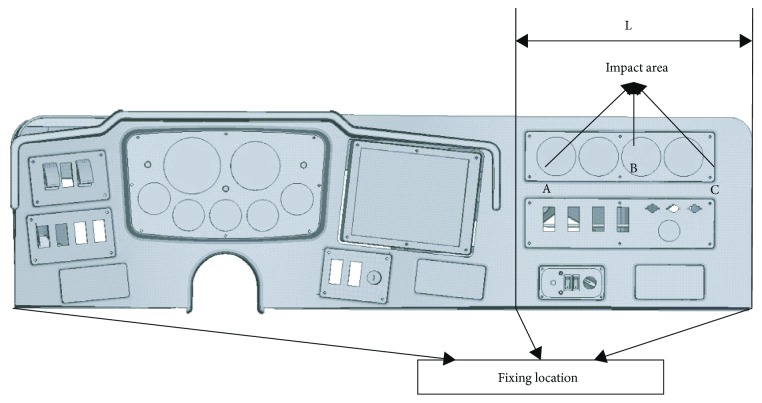
Impact area.

The impact location should cover the high aggressive structures in the panel, including the fragile places and high stiffness structures. According to this principle, the impact locations in the panel were chosen as shown in Figure 3. The selection of the impact location is also to consider the potential contact locations of the occupant during operation.

The working speed of the dump truck usually does not exceed 40 km/h due to the terrible working environment. Therefore, the speeds of 10 km/h, 24.1 km/h, 32 km/h, and 40 km/h were used in this study with a combining consideration of the impact speed required by ECE R21 (24.1 km/h).

#### 2.3.2. Parametric Study

Table 4 shows the information for different parametric studies. Firstly, a parametric study was carried out on the influence of the panel type (soft versus hard) on head injuries by comparing the results from the impact simulation of a soft instrument board with that from a hard panel. Then the effects of filling elastic modulus (filling E: 200 versus 20 MPa), frame elastic modulus (frame E: 0.34 versus 3.4 GPa), and fixing distance (L: 450 versus 550 versus 650 mm, see Figure 3) in the soft panel on head injuries were analyzed, respectively.

For the parametric study of softer panel design parameters, either different impact speeds (10 km/h, 24.1 km/h, 32 km/h, and 40 km/h) or different impact locations (A, B, and C) were used to consider the working environment of the dump truck. For the impact speed changing case impact, location B was selected to cover the main impact area (front of the occupant). While for the impact location changing case, impact speed of 24.1 km/h was used to take the requirement of ECE R21 (the only regulation for panel safety design) into consideration. The combinations of impact speed and location were not considered to reduce the computational time, and the increase of impact speed generally leads to a worse situation.

## 3. Results

### 3.1. Effects of Panel Type on Head Injury

**Figure 4:**
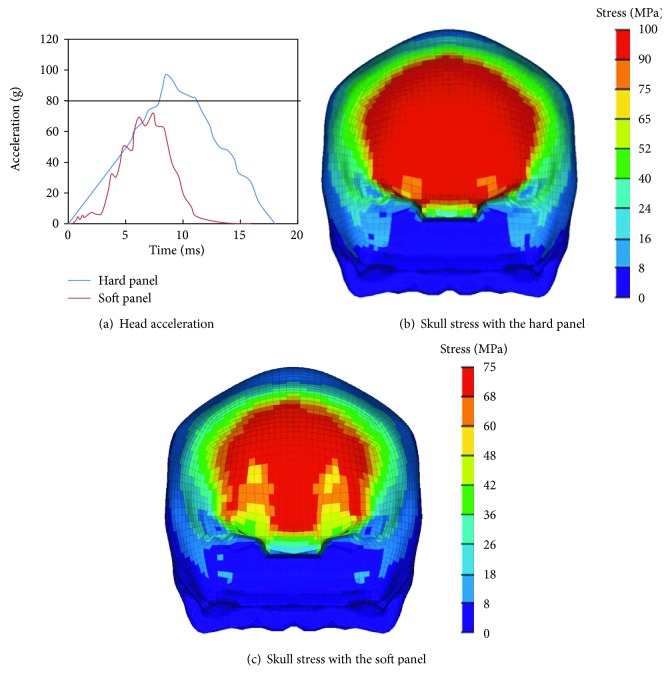
The simulation results for the hard and soft instrument panel.

The simulation results for different panel type are shown in Figure 4, including the time-acceleration curve and skull stress nephogram. The results show that the maximum acceleration of the head is 97 g (near 9.5 ms) for the hard panel, and the time range for head acceleration above 80 g in the hear panel case is from 7.6 ms to 13 ms (the peak width = 5.4 ms), see Figure 4(a). Both the peak value and duration time exceed the requirements in the ECE R21, where a maximum head acceleration of 80 g and a duration time of 3 ms are limited. However, for the soft panel, the maximum acceleration of the head is 72 g (at 7.4 ms), which meets the requirement of ECE R21. Similarly, the maximum stress of the skull for the impact with the hard panel (100 MPa) is significantly higher than that for the soft panel (75 MPa), and the peak stress area for the hard panel case is also obviously wider than that for the impact with the soft panel.

### 3.2. Effects of Soft Panel Design Parameters on Head Injury at Different Impact Speeds

**Figure 5:**
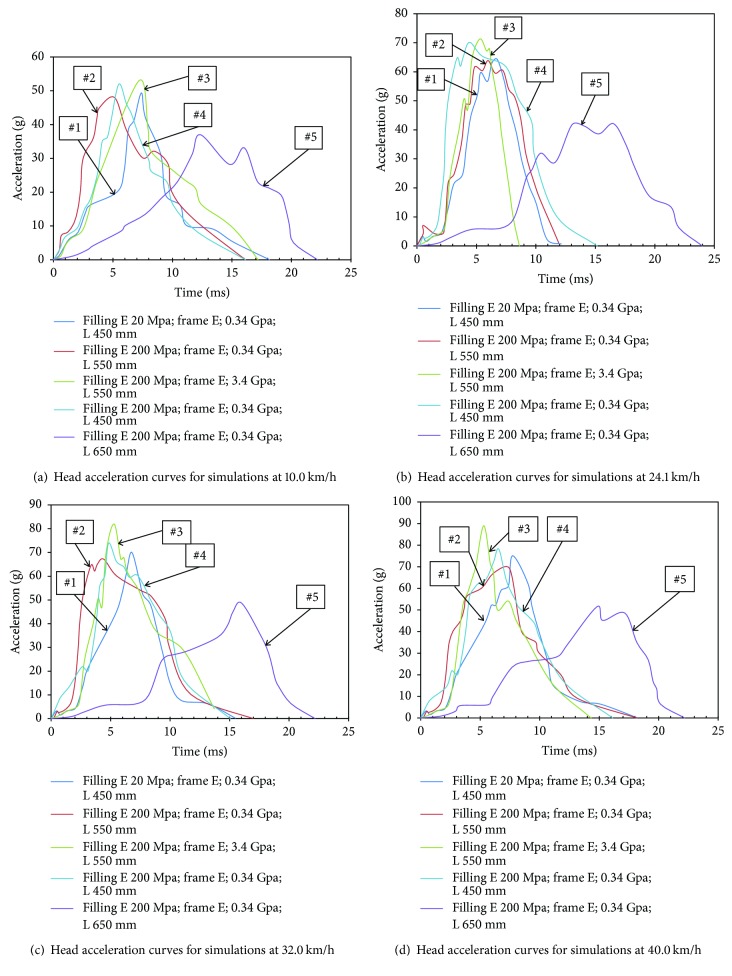
Head acceleration curves for simulations using different design parameters.

Figure 5 shows the head acceleration curves for simulations of head-to-soft panel impact using different design parameters at different speeds. As shown in this figure, the peak acceleration values of head for the cases of filling elastic modules of 20 MPa are significantly lower than those for the cases of using a filling elastic modules of 200 MPa (#1 versus #4: 50 g versus 52 g, 64 g versus 71 g, 70 g versus 74 g, and 75 g versus 78 g for 10 km/h, 24.1 km/h, 32 km/h, and 40 km/h, resp.), when keeping other design parameters at the same level. The maximum head acceleration for the impacts with a panel using a frame elastic modules of 0.34 GPa (#2: 63 g, 48 g, 67 g, and 69 g) are also obviously lower than those for a stiffer frame with elastic modules of 3.4 GPa (#2 versus #3: 48 g versus 53 g, 63 g versus 72 g, 67 g versus 82 g and 69 g versus 89 g for 10 km/h, 24.1 km/h, 32 km/h, and 40 km/h, resp.), again other design parameters were controlled. For different fixing distances (controlling other parameters), the results show that the head peak acceleration values for the cases of 450 mm are the highest, followed by the cases of 550 mm and 650 mm (#4 versus #2 versus #5: 52 g versus 48 g versus 37 g, 71 g versus 63 g versus 42 g, 74 g versus 67 g versus 48 g, and 78 g versus 69 g versus 51 g for 10 km/h, 24.1 km/h, 32 km/h, and 40 km/h, resp.).

**Figure 6:**
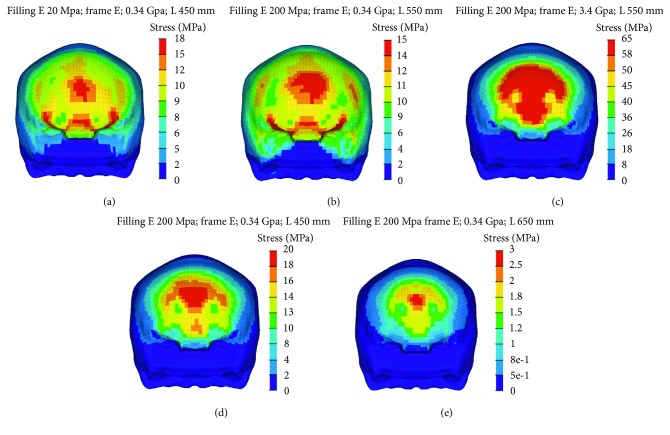
Skull stress for simulations at 24.1 km/h.

Figure 6 shows an example for the distribution of skull stress at the time point where the maximum stress occurred. These results show that the maximum stress value of the skull for the case using a filling elastic modulus of 20 MPa is slightly lower than that for the case with a filling elastic modulus of 200 MPa when controlling other parameters. The peak skull stress area for the 20 MPa filling elastic modulus case is also smaller than the 200 MPa case. However, significant differences in maximum skull stress and its area were observed between the cases using a frame elastic modulus of 0.34 GPa and 3.4 GPa, where the maximum skull stress for the former is 15 MPa (Figure 6(b)) and 65 MPa (Figure 6(c)) for the latter. The maximum skull stress for the fixing distance of 450 mm, 550 mm, and 650 mm are 20 MPa (Figure 6(d)), 15 MPa (Figure 6(b)), and 3 MPa (Figure 6(e)), respectively. The case of 650 mm has the smallest peak stress area in the skull comparing to another two cases.

**Figure 7:**
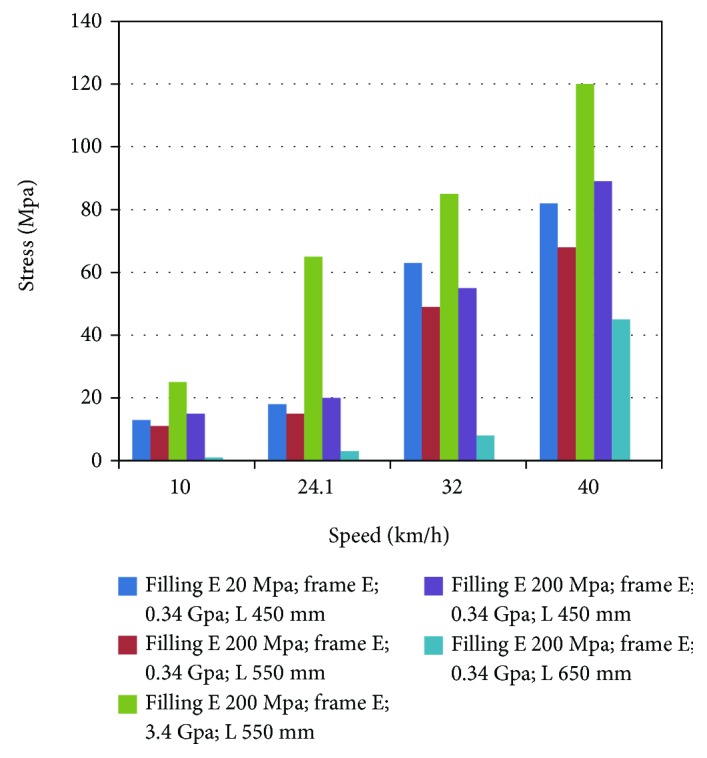
The peak value of the skull for simulations at different speed.

The stress distributions for the speeds of 10 km/h, 32 km/h, and 40 km/h are not shown here, but the similar (to the 24.1 km/h case) trend of the maximum stress as a function of changing the magnitude of a given design parameter was observed for other impact speeds (Figure 7). The data in Figure 7 also show that the maximum skull stress increases with increasing impact speed.

### 3.3. Effects of Soft Panel Design Parameters on Head Injury at Different Impact Locations

**Figure 8:**
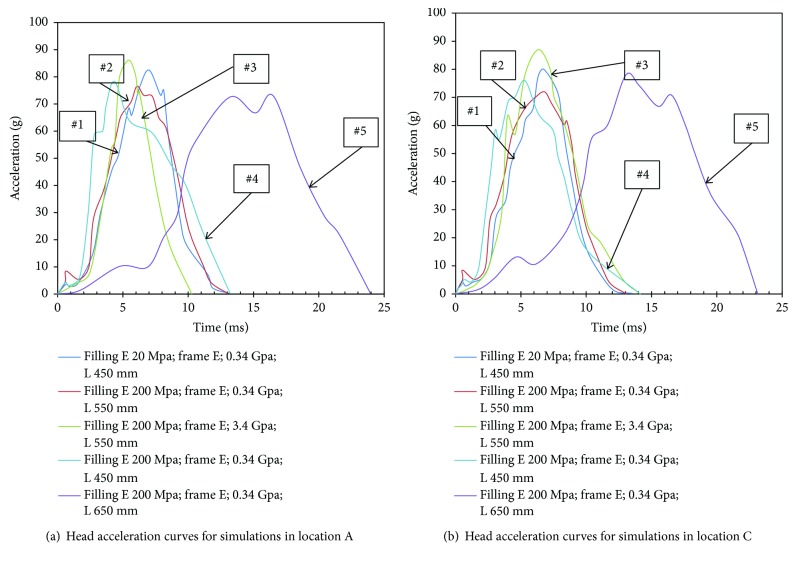
Head acceleration curves for simulations in different locations.

**Figure 9:**
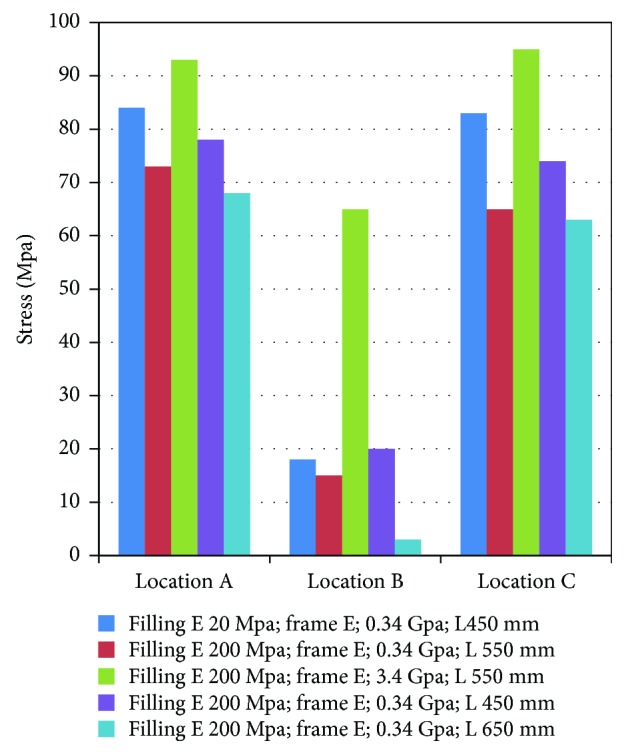
Skull stress of the simulation in location A, B, and C at 24.1 km/h.

Figures 8 and 9 show the predicted head acceleration time history curves and the maximum skull stress values for the simulations using different design parameters at different impact locations. It is clear from these data that both peak head acceleration and maximum skull stress are lower in the cases with a relatively softer panel and longer fixing distances. This trend is constant for all impact locations, which is similar to that observed from changing impact speed.

The simulation results also show that the peak head acceleration values and maximum skull fractures in locations A and C (Figures 8 and 9) are larger than those for the location B (Figures 5(b) and 9).

## 4. Discussion and Conclusions

The effects of different panel type and design parameters of the soft panel on head injury index of peak acceleration and maximum skull stress in head-to-truck panel impacts were predicted using FE simulations. Comparisons of head acceleration and skull stress between hard and soft panel impacts indicate that the soft panel is beneficial for head protection when impacting with a truck instrument panel. This effect is mainly from the generally lower stiffness of the soft panel.

For the detailed analysis of how different design parameters of the soft panel affect head injuries in the head-to-panel impact, the results indicate that a lower filling elastic modulus, lower frame elastic modulus, and longer fixing distance are helpful for head injury prevention in head-to-panel impacts. Moreover, the above trends are not affected by changing the impact location and speed to some extent. The results also suggest that the head peak acceleration and maximum skull stress are more sensitive to the fixing distance than elastic modulus of the filling. This is mainly due to the fact that increasing the fixing distance leads to a significant increase of panel deformation in the head-to-panel impact, which absorbed lot impact energy for head protection. While the differences in panel deformation from changing filling and frame elastic modulus are smaller than that from the change of fixing distance. Therefore, a safer panel design for head injury prevention is firstly suggested to have a long fixing distance and then followed by using softer filling and frame materials.

## Figures and Tables

**Table 1:** Materials of bones [6–8].

Material	Density (g/cm^3^)	Young's modulus (GPa)	Poisson's ratio	Shear modulus (GPa)	Hardening parameter	Cowper-Symonds model		Molding failure strain (%)
						C	P	
Cranial cortical bone	2.0	11.5	0.3	1.15	0.1	2.5	7	0.02
Cancellous bone of skull	1.0	0.04	0.45	0.001	0.1	2.5	7	0.03
Bone of cortical bone	5	21	0.23	1.15	0.1	2.5	7	0.02
Cancellous bone of facial bone	1	0.04	0.45	0.01	0.1	2.5	7	0.03
Mandibular cortical bone	2	11.5	0.3	1.15	.1	2.5	7	0.02
Mandibular cancellous bone	1	0.04	0.45	0.01	0.1	2.5	7	0.03

**Table 2:** Head soft tissue material parameters.

Head component	Density, *ρ* (kg/m^3^)	Young's modulus (MPa)	Poisson's ratio	G0 (kPa)	G∞ (kPa)	*β* (s-1)	Bulk modulus (MPa)	Reference
Scalp	1000	16.7	0.42					[9]
Dural	1130	31.5	0.23					[9]
Pia mater	1130	11.5	0.45					[9]
CSF cerebrospinal fluid	1050			100	20	100	4.97	[9]
Brain	1040			1.66	0.928	16.95	557	[10]
Cerebellum	1040			1.66	0.928	16.95	557	[10]
Brainstem	1040			1.66	0.928	16.95	557	[10]
Corpus callosum	1140	31.5	0.45					[11]
Sickle	1140	31.5	0.45					[11]
Pituitary/ventricles	1140	31.5	0.45					[11]

**Table 3:** The materials defined for dump truck frame and panel [4].

Structure	Material	Young's modulus (MPa)	Poisson's ratio	Density, *ρ* (kg/m^3^)
Framework	DC01	2.1 × 10^5^	0.28	7.85 × 10^3^
Hard panel	PC	2400	0.35	1007
Soft panel				
Skin	PVC	2400	0.35	1350
Frame	ABS	3400	0.35	1007
Filling	PUR	20.0	0.01	140

**Table 4:** The properties of the panel for different parametric studies.

Parametric study	Filling	Frame	L	Speed (km/h)	Location
Panel type	Soft versus hard (see Table 3 for material parameters)		450 mm	24.1	B

Filling elastic modulus	200 versus 20 MPa	0.34 GPa	450 mm	10, 24.1, 32, and 40 at B	A, B, and C at 24.1 km/h
Frame elastic modulus	200 MPa	0.34 versus 3.4 GPa	550 mm		
Support position	200 MPa	0.34 GPa	450 versus 550 versus 650 mm		
